# An Atypical Migration Pattern of Tattoo Pigment

**DOI:** 10.7759/cureus.40640

**Published:** 2023-06-19

**Authors:** Emily M Cao, Mckenzie DiLeo, Emelie E Nelson, Morgan A Rousseau, Rashid M Rashid

**Affiliations:** 1 Dermatology, University of Texas Health Science Center at Houston McGovern Medical School, Houston, USA; 2 Internal Medicine, University of Texas Health Science Center at Houston McGovern Medical School, Houston, USA; 3 Dermatology, Mosaic Dermatology, Houston, USA

**Keywords:** tattoo pigment spread, tattoo removal, tattoo blowout, tattoo complication, tattoo

## Abstract

Unwanted diffusion of pigment past the original margins of a tattoo, termed “tattoo blowout,” appears as a blurred rim classically surrounding the original tattoo borders. The darkened skin is visibly noted within hours to days following the tattooing procedure. Although this complication is not largely covered in literature, in prior cases, blowout has been shown to occur in areas of thin skin such as the dorsum of the foot. We present a rare presentation of tattoo blowout in a possible gravity-dependent pattern of pigment migration, occurring years after tattoo application. This atypical presentation highlights the knowledge gap that exists in the medical literature surrounding the mechanism of tattoo blowout and reinforces that physicians should be aware of these potentially poor aesthetic outcomes.

## Introduction

“Tattoo blowout” is an acute phenomenon that refers to the undesired spread of pigment beyond the original margins of the tattoo, often resulting in a “blurry halo” border [1]. The “blowout” characteristically presents as circumferential hyperpigmentation surrounding the original tattoo. Tattoo blowout has been reported as early as one day following tattoo application [2,3] upto during the healing phase [1]. This complication has been correlated to unintentional injection of pigment into subcutaneous fat, improper tattooing technique, and impaired wound healing, often occurring in areas of thin skin such as the dorsal foot [1]. We present a case of delayed pigment migration limited to the inferior border of a tattoo, in a seemingly gravity-dependent pattern.

## Case presentation

**Figure 1:**
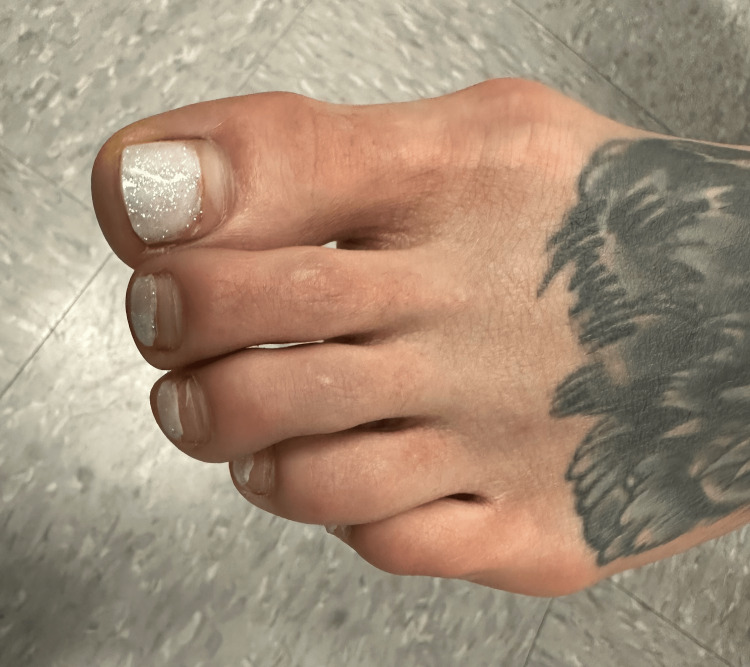
Dorsal left foot with pigment migration from the inferior tattoo border

A 30-year-old female presented to dermatology for the evaluation of a dorsal foot tattoo that she obtained five years prior. The tattooing procedure was without complications and healed as expected; however, a few years later, the patient noticed unsightly discoloration of nearby non-tattooed skin. While the patient was concerned about the tattoo’s physical appearance, she denied the presence of pain or any other associated symptoms. Furthermore, the patient reported no significant past medical or dermatologic history. A physical exam revealed hyperpigmentation beginning near the inferior border of the tattoo and extending toward the metatarsophalangeal joints (Figure 1). Notably, the surrounding lateral and superior skin visually remained unaffected. This case represents the first reported case of tattoo blowout in a visibly gravity-dependent pattern of pigment migration.

## Discussion

Tattoo blowout refers to the migration of tattoo pigment beyond the original margins of the tattoo. Unlike the characteristic circumferential spread of a typical tattoo blowout, our patient presented with a subcutaneous spread only beyond the inferior margin of the tattoo. Furthermore, our patient presented with her complaint five years after receiving her tattoo, having only observed the abnormal pigment migration years afterward. Our patient’s presentation, both in terms of the appearance and delayed nature of the presentation, is unique. While the mechanism of tattoo blowout is not completely understood, gravity, improper tattooing technique, and lymphatic or blood-borne spread of pigment have been proposed.

Because the dermis of the dorsal skin is relatively thin, it is possible that inappropriate placement of pigment into the hypodermis during tattoo application may contribute to tattoo blowout, a mechanism that was proposed in a prior case of tattoo blowout that histologically confirmed the presence of pigment in the dermis and subcutaneous fat [3]. However, it should be noted that in that case, the tattoo blowout was observed within one day of tattoo application. In contrast, our patient’s blowout occurred years later. 

Lymphatic or blood-borne dispersion of tattoo ink has also been proposed as a mechanism of tattoo blowout. In a 2017 study, tattoo pigment deposits were found in the skin, lymph nodes, and Kupffer cells in tattooed mice [4]. However, no deposits were found in internal organs, findings that could support a blood-borne lymphatic etiology.

A visual inspection might instead suggest gravity as a contributing factor. The tattoo was performed on the dorsal foot and the pigmentary changes extended only beyond the inferior border. This pattern follows the natural downward curvature of the dorsal foot toward the metatarsophalangeal joints. The gravitational hypothesis also aligns with the extended timeline before presentation, as this mechanism would possibly require repetitive use of the foot over years for the pigment to descend. It is possible that the tattoo pigment traveled into the interstitium and migrated through interstitial fluid.

Because tattoos are becoming increasingly common, it is important to address potential complications that may arise. Although a tattoo blowout is asymptomatic, patients may be displeased with the appearance of the pigment migration. Treatment options include additional tattooing over the area of discoloration or laser therapy with lasers such as the Q-Switched Nd:YAG laser [2,3]. However, laser tattoo removal may be costly and can take multiple sessions, typically six to eight weeks apart [2].

## Conclusions

We present the first case in the literature of an atypical pigment migration solely on the inferior border of a dorsal foot tattoo. Cases of “tattoo blowout” share characteristics with this presentation; however, pigment migration is often observed across all tattoo margins shortly after placement. Our patient presented with an inferior spread of pigment years after the tattoo application. While the exact cause of this presentation is unknown, lymphatic drainage, gravity, or laxity in aftercare could contribute to this abnormal diffusion. This case adds dimension to our knowledge of potential deviations in tattoo pigment distribution. It is important that physicians are aware of possible acute complications so that they can advise patients of potential risks or undesired aesthetic outcomes.
